# An Electrochemical DNA Biosensor Developed on a Nanocomposite Platform of Gold and Poly(propyleneimine) Dendrimer

**DOI:** 10.3390/s8116791

**Published:** 2008-11-01

**Authors:** Omotayo Arotiba, Joseph Owino, Everlyne Songa, Nicolette Hendricks, Tesfaye Waryo, Nazeem Jahed, Priscilla Baker, Emmanuel Iwuoha

**Affiliations:** SensorLab, Department of Chemistry, University of the Western Cape, Bellville, Cape Town 7535, South Africa; E-mails: oarotiba@uwc.ac.za; jowino@uwc.ac.za; esonga@uwc.a.za; nhendricks@uwc.ac.za; twaryo@uwc.ac.za; njahed@uwc.ac.za; pbaker@uwc.ac.za

**Keywords:** Poly(propyleneimine) dendrimer, gold nanoparticle, electrochemical impedance spectroscopy, electrochemical DNA biosensor, DNA

## Abstract

An electrochemical DNA nanobiosensor was prepared by immobilization of a 20mer thiolated probe DNA on electro-deposited generation 4 (G4) poly(propyleneimine) dendrimer (PPI) doped with gold nanoparticles (AuNP) as platform, on a glassy carbon electrode (GCE). Field emission scanning electron microscopy results confirmed the co-deposition of PPI (which was linked to the carbon electrode surface by C-N covalent bonds) and AuNP ca 60 nm. Voltammetric interrogations showed that the platform (GCE/PPI-AuNP) was conducting and exhibited reversible electrochemistry (E°′ = 235 mV) in pH 7.2 phosphate buffer saline solution (PBS) due to the PPI component. The redox chemistry of PPI was pH dependent and involves a two electron, one proton process, as interpreted from a 28 mV/pH value obtained from pH studies. The charge transfer resistance (R_ct_) from the electrochemical impedance spectroscopy (EIS) profiles of GCE/PPI-AuNP monitored with ferro/ferricyanide (Fe(CN)_6_^3-/4-^) redox probe, decreased by 81% compared to bare GCE. The conductivity (in PBS) and reduced R_ct_ (in Fe(CN)_6_^3-/4-^) values confirmed PPI-AuNP as a suitable electron transfer mediator platform for voltammetric and impedimetric DNA biosensor. The DNA probe was effectively wired onto the GCE/PPI-AuNP via Au-S linkage and electrostatic interactions. The nanobiosensor responses to target DNA which gave a dynamic linear range of 0.01 - 5 nM in PBS was based on the changes in R_ct_ values using Fe(CN)_6_^3-/4-^ redox probe.

## 1. Introduction

A biosensor is an analytical device that incorporates a bioreceptor onto a transducer surface and in the presence of an analyte, produces measurable signals (due to a bio-recognition event) that are proportional to the analyte concentration. Biosensors can be classified using either the bioreceptor or the transduction method or both. The common bioreceptors are enzyme [1-4], antibody [5, 6], DNA [7, 8] and whole cell [9], while transduction methods include electrochemical, pizoelectric, optical, etc. [5, 10]. Biosensors exploit the excellent selectivity, specificity and reactivities of immobilized biomaterials towards their substrates. They are relatively less expensive, faster, more user friendly and miniaturizable, compared to traditional instrumental methods. Owing to these unique qualities, biosensors are increasingly being applied in biomedical and environmental analysis.

Recently, Mascini and co workers [11] reported an electrochemical DNA biosensor for the detection of low-molecular-mass substances that are of environmental concern, including polychlorinated biphenyls (PCBs) and aflaxtoxins. Other researchers have reported environmental biosensor systems for the detection of organophosphates in air, soil and water [12], pesticides in soil [13], water pollutants [14, 15], food contaminants [16, 17], explosives [18] and chemical warfare agents [19]. Despite the milestones achieved in biosensor development and applications, there still remains the challenge of achieving lower detection limits, greater sensitivity, biocompatibility and reproducibility [20]. The step in biosensor preparation that has the greatest impact on biosensor performance is the immobilization of the biomolecular probe. The two routes usually adopted to optimize the immobilization of biomolecules are chemical modification of the substrate and biological functionalization of the biomolecule [21], both of which determine the immobilisation chemistry. Lucarelli *et al.* [8] and Sassolas *et al.* [22] demonstrated in their studies how the functionalization of oligonucleotides improves their immobilization chemistry and hence the performance of DNA biosensors. On the other hand, the modification of electrodes or substrates using nanomaterials such as metal oxide nanoparticles [23, 24] and polymers [25] are now emerging. In this study, the functionalization of both the immobilization platform and the DNA will be performed with the aim of improving the biosensor response characteristics. This involves the use of GCE modified with dendrimeric materials that are doped with gold nanoparticles. Dendrimers are synthetic three-dimensional macromolecules with a well-defined, highly branched and globular shaped molecular structure [26]. Poly(propyleneimine) (PPI) and other dendrimers such as poly(amidoamine) (PAMAM) are widely used in biomedical research in the form of nanoscopic containers for genes and drugs [27-29]. Dendrimers have also been used in catalysis [30, 31] and very recently in biosensors [32, 33] and microbicides [34, 35].

Gold nanoparticles (AuNP) are known to exhibit excellent biocompatibility and high conductivity. They act as bimolecular nanoscopic wires that create large electrode surface areas that suitably orient DNA molecules for optimal immobilization, and have been used to chemisorb thiolated DNA onto electrode surface [36-42]. This paper describes the preparation and electrochemical responses of a DNA nanobiosensor consisting of AuNP-doped PPI and thiolated 20mer oligonucleotide immobilized on a GCE.

## 2. Results and Discussion

### 2.1 Morphology and Voltammetric behaviour of GCE/PPI-AuNP

**Figure 1.**
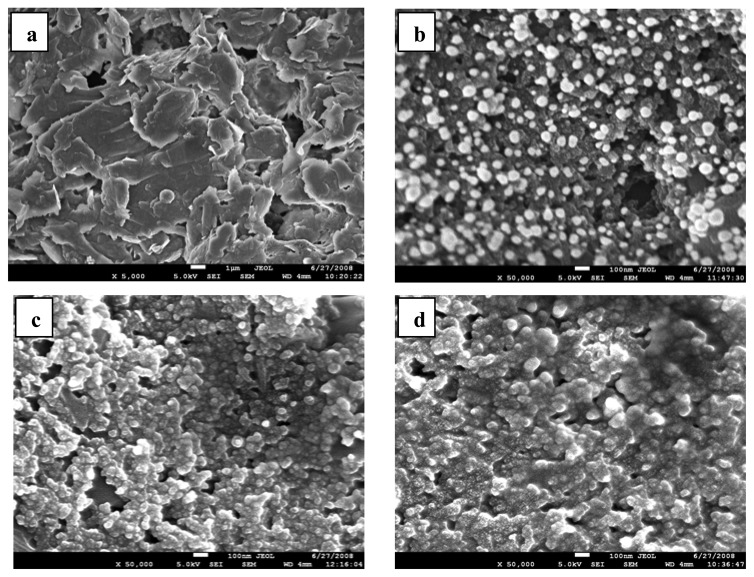
FE-SEM images on screen printed carbon electrodes (SPCE) (a) blank SPCE. (b) SPCE/AuNP. (c) SPCE/PPI (d). SPCE/PPI-AuNP.

Figures 1a-d show the FE-SEM images of the blank SPCE, SPCE/AuNP, SPCE/PPI and SPCE/PPI-AuNP, respectively. An average size of 60 nm AuNP can be observed deposited on the surface of the SPCE in Figure 1b (compare with blank SPCE in Figure 1a). Figure 1c confirms the attachment of PPI onto the carbon surface, seen as a globular growth on the SPCE. At the point of measurement, Figure 1d exhibited a reflectance not observed in Figure 1c as a result of the AuNP which was co-deposited. The diameter of G4 PPI is about 3.12 nm [43] and has been known to be flexible thus in Figure 1d,

PPI appears to cluster around the AuNP because it is smaller in size. The nanocomposite however has higher particle size than the separate components; the morphology of the PPI-AuNP is similar to that of PPI only (Figure 1c). As a further proof of the presence of AuNP in Figure 1d, energy-dispersive x-ray analysis (EDAX) of the sample gave 4.95 weight percent of gold relative to carbon and other elements present. The TEM image of GCE/PPI-AuNP gave a smaller AuNP size of about 30 nm (result not shown) and this suggests that the roughness of the electrode (substrate) has an effect on the AuNP distribution and particle size.

**Figure 2.**
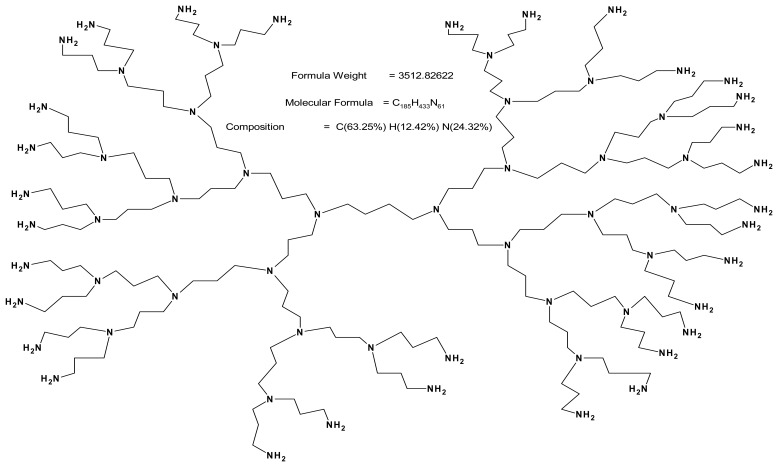
Structure of G4 Poly(propylene imine) dendrimer showing the peripheral primary amine and internal tertiary amine.

The chemical (covalent) modification of GCE using either aliphatic or aromatic primary amines to form C-N bonds has been in use for quite a while and its mechanism involves the formation of an amine cation radical [44-46]. This reaction mechanism has not been known to occur with tertiary amines [45]. G4 PPI consists of peripheral primary amines and internal tertiary amines. Thus the same chemistry should apply in the attachment of the peripheral primary amines (and not the internal tertiary amines) of PPI (Figure 2) onto the GCE (also SPCE for SEM) surface at a potential of ca 1000 mV where electro-oxidation of primary amines [45] occur. Cyclic voltammetric deposition of PPI and AuNP on the GCE, therefore, involves the formation of amine linkages between PPI and GCE while the AuNP were simultaneously deposited with the PPI.

**Figure 3.**
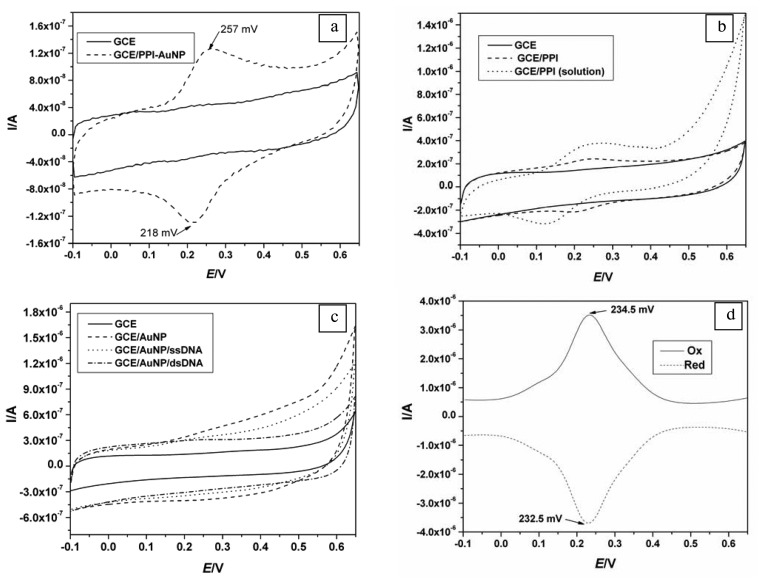
**(a)** CV of GCE and GCE/PPI-AuNP in PBS from -100 mV to 650 mV at 20 mV/s. **(b)** CV of 3 mM PPI solution on GCE and GCE/PPI. Background electrolyte is 10 mM PBS. **(c)** CV of GCE and GCE/AuNP with ssDNA and dsDNA in PBS. **(d)** Oxidative and reductive square wave voltammograms of GCE/PPI-AuNP in PBS.

Figure 3a, compares the electrochemical behaviour of GCE/PPI-AuNP (dotted line) against the bare GCE (solid line) in PBS. The PPI-AuNP composite film exhibited reversible electrochemistry characteristic of surface adsorbed species with formal potential E^0^′ = 233 ± 5 mV for six different measurements demonstrating the good reproducibility of the composite platform. To ascertain the species responsible for the reversible peaks, PPI and AuNP were deposited alone (Figures 3b and 3c). In Figure 3b, 3 mM PPI in PBS solution exhibited a quasi-reversible electrochemistry and when it was electro-deposited, the anodic and cathodic peaks separation became less. In Figure 3c, where only AuNP was deposited on GCE, no peaks were observed. This meant that the pair of peaks observed in Figure 3a (dotted line) was due to the PPI component of the nanocomposite. The reversibility of the electrochemical oxidation/reduction occurring within the PPI-AuNP nanocomposite platform was confirmed by the ratio of anodic (I_pa_) to cathodic (I_pc_) peak currents, which was calculated to give 0.992. Also the anodic and cathodic square wave voltammograms gave approximately the same peak potential value (see Figure 3d). In addition, the integration of the anodic and cathodic CV peaks from Figure 3a (wave GCE/PPI-AuNP) gave charges of 528.1 nC and -524.7 nC, respectively, which are the same within experimental error.

**Figure 4.**
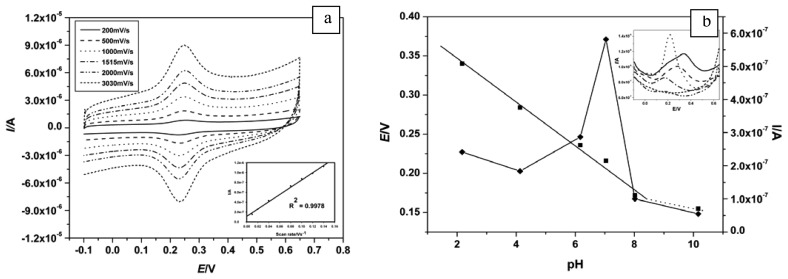
**(a)** CV of the GCE/PPI-AuNP in PBS as a function of scan rate (inset is the scan rate dependence of I_pa_). **(b)** Plot of E_pa_ and I_pa_ vs pH and SWV (inset) response of platform in 0.1 M PBS ant different pH.

Figure 4a shows the CV of GCE/PPI-AuNP at different scan rate in PBS. From this figure, (i) the currents increased with increase in scan rate with no shift in potential, (ii) I_pa_ was proportional to scan rate and (iii) a plot of I_pa_ versus scan rate showed linearity (Figure 4a inset) with a correlation coefficient of 0.9978. It can thus be deduced that the platform was conducting and exhibited a reversible electrochemistry characteristic of surface adsorbed specie because I_pa_ versus scan rate was linear. Ideally, for surface adsorbed specie, E_pa_ should be the same as E_pc_. However, the AE of ca 30 mV observed here may be as a result of diffusion of electrons along the PPI matrix and suggests a two electron system. The fact that there was no shift in potential and the I_pa_/I_pc_ remained unity also showed the stability of the PPI-AuNP platform in PBS.

### 2.2 pH studies of GCE/PPI

The PPI on the electrode consists of secondary and tertiary amine molecules which are responsible for its reaction. PPI are positively charged (cationic) polyelectrolyte in their protonated form. The ionization behavior of PPI has been studied using potentiometry and NMR. These studies [48, 49] revealed that PPI can be protonated to a degree of 2/3. For more insights into the number of electron(s) and proton(s) involved in the electrochemistry of immobilized PPI, effect of variation in pH on its voltammetric response was studied in PBS. From the CV (Table 1) and SWV (Table 1 and Figure 4b) data, it can be inferred that the optimum pH for the PPI's reversibility and conductivity is ca. 7. Figure 4b shows the plot of E_pa_ and I_pa_ vs pH obtained from SWV (Figure 4b inset). E_pa_ shifted cathodically (to lower values) as pH increased from 2 to 8 (Figure 4b solid line (■)) following the relationship *E_pa_* = 399.97 −0.028*pH* (and *E*°’ = 425.74 − 0.030*pH* for CV). The 28 mV per pH unit, which is close to the Nernstian value of 29.5 mV (59/n for n =2), showed that the redox mechanism of PPI involves a two electron, one proton process [50]. Above the pH of 8.5 there was a deviation from this relationship and the electrochemistry was completely quenched at pH 12. From this response to pH, it can be inferred that the electrochemistry of the nanocomposite is facile only when the dendrimer is moderately protonated. The pKa of tertiary amines is ca. 10 (pKb = 4) thus, the common rules (derived from Henderson-Hasselbach equation) which states that (i), at *pH* ≤ *pKa*–2 the substance exists mostly in its associated or protonated form and (ii), at *pH* ≥ *pKa* + 2 the substance exists mostly in its dissociated or deprotonated form, supports our observation. The dendrimer can be thought to be practically completely protonated up to the pH of 8.5 and below and totally deprotonated above pH 12. The pH dependence of PPI observed agrees with Koper and co-workers [51], who carried out a ^15^N-NMR study of PPI using Ising model. They observed variations in the chemical shifts of tertiary nitrogens with pH as a result of protonation, which vary in degree from shell to shell in a two step mechanism.

### 2.3 Electrochemical Impedance spectroscopy of GCE/PPI-AuNP

**Figure 5.**
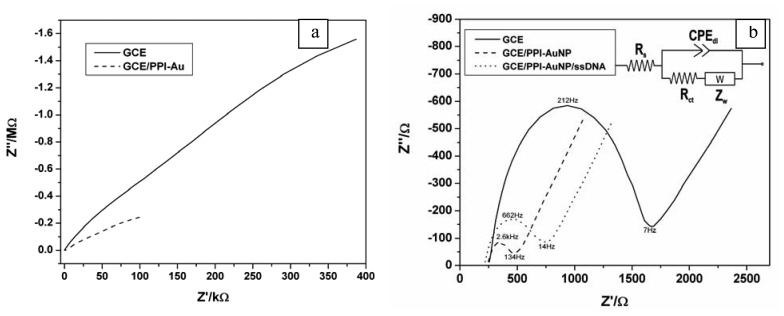
**(a)** Nyquist plot of bare GCE and GCE/PPI-AuNP in PBS. **(b)** Nyquist plot of GCE, GCE/PPI-AuNP and GCE/PPI-AuNP/ssDNA in 5 mM Fe(CN)_6_^3-/4-^ redox probe.

Charge transfer resistance (in form of a semi circle) was not observed in the complex plane plot of the GCE/PPI-AuNP in PBS (Figure 5a). This observation is expected for a reversible system where the charge transfer is very facile (as seen in the high scan rates in Figure 4a) hence R_ct_ ≈ R_s_. Thus EIS also confirmed the reversible electrochemistry of PPI-AuNP as discussed with voltammetry. Total impedance (in PBS) of GCE/PPI-AuNP was remarkably lower than that of bare GCE, confirming the presence of a conducting layer. Figure 5b shows the complex plane impedance behaviour of GCE/PPI-AuNP electrode system in Fe(CN)_6_^3-/4-^ redox probe while Table 2 shows the equivalent circuit (Figure 5b inset) parameters of GCE/PPI-AuNP at 200 mV. The E°’ and lowest R_ct_ of Fe(CN)_6_^3-/4-^ redox chemistry on GCE [33] and GCE/PPI-AuNP (not shown) occurred at ca 200 mV, hence the choice of 200 mV as the bias potential in the EIS studies. The Fe(CN)_6_^3-/4-^ redox probe exhibited kinetic control and diffusion controlled electrochemistry at high and low frequency respectively at the PPI-AuNP interface. Complex phase element, CPE, was used in the model because the semi circle observed was depressed, the phase angle observed in the measurement was less than 90° (not shown) and CPE is also appropriate to model the non ideal behaviour of the inhomogeneous electrode surface. Solution resistance, R_s_, was used to model the electrolyte resistance at high frequency when the double layer capacitance is very small and the charge transfer kinetics is just at the onset. Charge transfer resistance, R_ct_, corresponding to the diameter of the semicircle in Figure 5b was used to model the resistance of the Fe(CN)_6_^3-/4-^ redox probe at the electrode surface. At a sufficiently low frequency, diffusion controlled process becomes limiting and this was modelled by Warburg impedance (Z_w_).

From Figure 5b, R_ct_ for GCE/PPI-AuNP decreased by 81% compared to that of the bare GCE. The positively charged (cationic) platform (AuNP is Au°) attracted the negatively charged Fe(CN)_6_^3-/4-^ to its surface and facilitated the electron exchange for Fe(CN)_6_^3-/4^. The result showed that the redox kinetics of Fe(CN)_6_^3-/4-^ was faster when GCE was modified with PPI-AuNP and this makes the platform more suitable for biosensor redox mediation in the presence of Fe(CN)_6_^3-/4-^ redox probe. The decreased R_ct_ also suggests the improved conductivity. The PPI-AuNP can be said to have catalytic effect on the rate of the charge transfer kinetics or the faradaic process of the redox probe. This can be shown by calculating the time constant of the Faradaic process using the frequency (ω_max_) at the maximum imaginary impedance according to Eqns. 1a and b [47]:

(1a) $$\omega_{\max}=\frac{1}{R_{ct}C_{dl}}$$

(1b) $$\tau =R_{ct}C_{dl}$$

where C_dl_ = double layer capacitance; τ = time constant; *ω*_max_ = 2*πf*

From Table 2, for the bare GCE, R_ct_ = 1348 Ω, *ω*_max_ =2*πx*212*Hz* Therefore C_dl_ = 0.557 μF and τ = 7.508 × 10^-4^ s rad^-1^. For GCE/PPI-AuNP, R_ct_ = 251.2 Ω, *ω*_max_ = 2*πx*2.07*kHz.* Therefore C_dl_ = 0.306 μF and τ = 7.687 x 10^-5^ s rad^-1^. The time constant values showed that the Faradaic process of the Fe(CN)_6_^3/4-^probe is one order of magnitude faster on PPI-AuNP-modified GCE than on bare GCE, which confirms the catalytic effect of the PPI-AuNP film. Exchange current *i*_0_, was used as a measure of the rate of electron transfer on the bare and modified GCE's. The *i*_0_ of the electrode systems is given by

(2) $$i_{0}=\frac{RT}{nFR_{ct}}$$

where R, F and n are gas constant, Faraday constant and number of electrons transferred, respectively. The *i*_0_ values for the electron transfer reaction of Fe(CN)_6_^3-/4-^ on bare GCE and GCE/PPI-AuNP are 1.905 x 10^-5^ A and 1.022 x 10^-4^ A, respectively. Control experiments that were performed to check the behavior of the Fe(CN)_6_^3-/4-^ probe on GCE/PPI (without Au-NP) gave *i*_0_ values that were lower than what was obtained with GCE/PPI-AuNP. Thus, some possible reasons for the catalytic effect (increase in reaction rate) observed at the GCE/PPI-AuNP/ Fe(CN)_6_^3-/4-^ interface are: (i) nanostructured nature of the electrode surface, (ii) the enhanced surface area and conductivity due to AuNP, and (iii), an increase in Fe(CN)_6_^3-/4-^ flux to the electrode surface due to the electrostatic attraction between the cationic platform and the anionic Fe(CN)6^3-/4-^. A similar effect of improved electrochemical properties of Fe(CN)6^3-/4-^ using AuNP has been reported [52]. The ability of the PPI-AuNP platform to catalyze the Fe^3+^/Fe^2+^ redox reaction is a promising feature for redox mediation in enzymes which have Fe at the heme group. This catalytic effect was observed during the EIS measurements. It took 56 s to reach the 7 Hz (low frequency end of the charge transfer resistant) for GCE, whereas it took 29 s to reach 134 Hz frequency for GCE/PPI-AuNP.

### 2.4 The voltammetric responses of the biosensor

**Figure 6.**
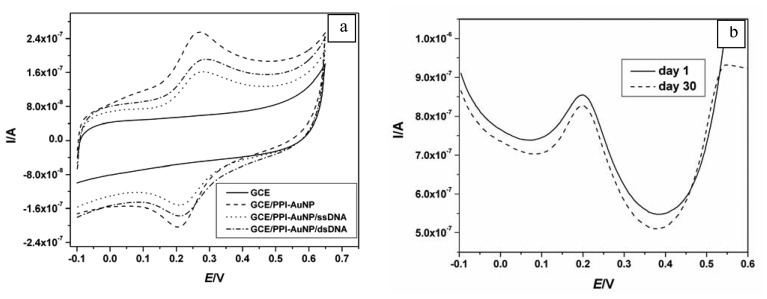
**(a)** CV of GCE/PPI-AuNP/ssDNA (developed with 2 μM thiolated ssDNA) and GCE/PPI-AuNP/dsDNA (i.e. response to 0.5 nM DNA target ssDNA) in PBS at 20 mV/s. **(b)** Differential pulse voltammograms of GCE/PPI-AuNP/ssDNA biosensor in PBS when stored for 30 days.

**Scheme 1.**
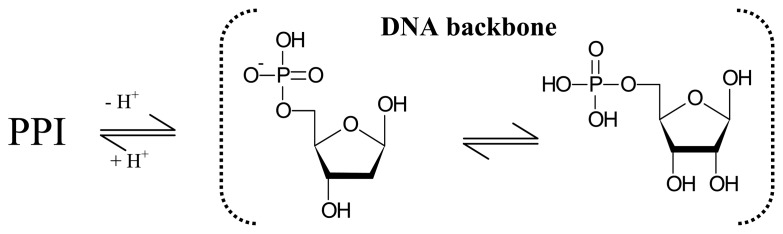
Proposed charge transfer scheme between the PBS electrolyte, DNA and PPI-AuNP.

Apart from AuNP enhancing the R_ct_ of Fe(CN)_6_^3-/4-^[52], AuNP was also incorporated into the composite for the purpose of connecting the thiolated probe ssDNA to the GCE surface via a Au-S linkage [37, 41]. The probe immobilization effectiveness would also be improved by an electrostatic attraction between the cationic platform and the anionic DNA probe. Figure 6a presents the cyclic voltammetric responses of the bare GCE, GCE/PPI-AuNP (the platform), GCE/PPI-AuNP/ssDNA (the biosensor) and GCE/PPI-AuNP/dsDNA (the hybridized biosensor). The biosensor stability monitored over a period of 30 days shown in Figure 6b, indicates effective adsorption of the probe DNA on the PPI-AuNP platform. As can be seen in Figure 6a, there was a 36% attenuation of the anodic peak current (I_pa_) of GCE/PPI-AuNP after the immobilization of the target ssDNA. However, the I_pa_ increased by 20% after exposing the resulting DNA biosensor to 0.05 nM target ssDNA (hybridization). This phenomenon may be attributed to the electrical or charge transportation properties of DNA. Various researchers [53-57], have shown that DNA charge-transfer characteristics can be explained on the basis of the two most fundamental processes for electron transfer in extended electronic systems, which are coherent tunnelling and diffusive thermal hopping. Furthermore, DNA's ability to undergo electron transfer and its conductivity are due to its ability to adopt different structures along the molecule as well as the polyelectrolyte character of the double helix, which may lead to the flow of positively charged counter ions along the negatively charged phosphate backbone, with electrons and holes appearing to shuttle along a single DNA molecule over a distance of a few nanometres. While most of these views are based on the bases (i.e. guanine, cytosine, adenosine and thymine) in DNA, electron delocalization in the conducting band through the phosphate backbone has also been proposed [58]. Based on these theories of DNA behaviour, the electrochemical responses of the GCE/PPI-AuNP after probe ssDNA immobilization and hybridization with target ssDNA can be attributed to the possibility of charge transfer between the cationic PPI and DNA base stacks and/or anionic backbone. Though DNA is not electroactive at +230 mV unlike the PPI (Figure 3c), its 2-deoxy-riboso-5-phosphate backbone, provides with PPI a supramolecular setting in which protons can be delocalized over a wider space and their contribution could be under potential control. Earlier studies [55] have shown that PPI can be an efficient hydrogen donor. It can therefore be speculated that DNA's conductivity allowed a certain degree of protonation of PPI (Scheme 1).

Also the fact that electron or charge is able to tunnel through the DNA base stack can also lead to a delocalized electron flow between the DNA and PPI molecules. The increase in the number of the more conducting guanine-cytosine (G-C) base pairs as a result of the formation of dsDNA is responsible for the increased current when the biosensor was hybridized.

### 2.5 Impedimetric responses of the biosensors

**Figure 7.**
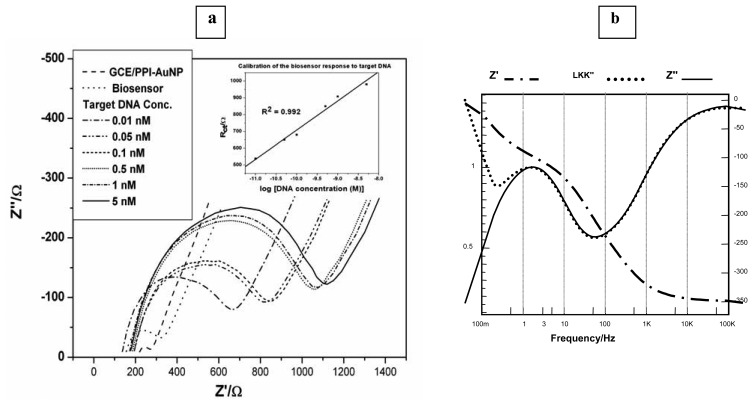
**(a)** Nyquist plots of the biosensor responses to 0.01 nM to 5 nM of target DNA in the presence of Fe(CN)_6_^3-/4-^ redox probe; **(b)** Kramer-Kronig (KK) plot for data validation. Z′ = experimental real impedance; LKK” = imaginary impedance calculated with the Kramer-Kronig equation; and Z” = the experimental imaginary impedance.

During the analysis of the spectra in Figure 5b, it was observed that R_ct_ increased by 276.4 Ω (Table 2) when the probe DNA was immobilised. The reason for this is that the negatively charged phosphate backbone of the single strand DNA attached to the platform repels the anionic Fe(CN)_6_^3-/4-^ redox probe. As shown in Figure 7a, after the pairing up (hybridisation) with target DNA, the anionic density of the resultant GCE/PPI-AuNP/dsDNA further increased the barrier for interfacial electron transfer because of the double strands formed, which further repelled the negatively charged Fe(CN)_6_^3-/4-^ redox probe and thus increased the R_ct_[60]. The Kramers Kronig transform (Eqn. 3) was used to validate the impedimetric responses shown in Figure 7. This integral equation allows the imaginary impedance, Z”, to be calculated from the real impedance, Z’, data. The experimental and calculated imaginary impedance show very good correlation as seen in Figure 7b.

(4) $$Z^{\prime \prime }\left(\omega \right)=-\frac{2\omega }{\pi }\int_{0}^{\infty }\frac{Z^{\prime }\left(x\right)-Z^{\prime }\left(\omega \right)}{x^{2}-\omega^{2}}dx$$

The impedance spectra in Figure 7a were fitted to the equivalent circuit in Figure 5b (inset) where the parallel R_ct_ and CPE were used to model the combination of the three layers, namely, the platform, probe DNA and target DNA. As explained earlier, the absence of a second semicircle is due to the fast redox of the platform chemistry. Hence, the R_ct_ of Fe(CN)_6_^3-/4-^ reports the DNA/ Fe(CN)_6_^3-/4-^ interfacial kinetics. Table 3 shows the values obtained from the circuit fitting. Fitting errors were less than 1% for R_ct_, which was chosen as the analytical parameter and less than 10% (not shown) for other circuit elements. Figure 7a inset shows the calibration plot of the DNA biosensor. A correlation coefficient, R^2^ = 0.992 and a sensitivity of 174 Ω/log M were obtained.

## 3. Experimental

### 3.1 Materials

Ultra pure water with resistivity 18.2 MΩ was prepared using a Millipore Synergy water purification system. All reagents were of analytical grade. Oligonucleotides (referred to as DNA) of 20 bases were supplied by Inqaba Biotechnical (Pty) Ltd, Cape Town, South Africa. The probe and complementary DNA's were SH-5′-AAGCGGAGGATTGACGACTA-3′ and 5′-TAGTCGTCAATCCTCCGCTT-3′, respectively. Generation 4 (G4) poly(propyleneimine) dendrimer was purchased from SyMO-Chem, Eindhoven, Netherlands. HAuCl_4_ was obtained from Sigma Aldrich and used as received.

### 3.2. Solutions

Phosphate buffer saline solution (PBS) of pH 7.2 was prepared with 10 mM of Na_2_HPO_4_, KH_2_PO_4_ and 0.3 mM KCl, while the non-saline was without KCl and is simply referred to as phosphate buffer solution. Five mM (1:1) solution of K_3_Fe(CN)_6_ and K_4_Fe(CN)_6_ ([Fe(CN)_6_]^3-/4-^) was prepared in 10 mM phosphate buffer solution (100 mL) at pH 7.2. For pH studies, 0.1 M phosphate buffer solutions of pHs ranging from 2 to 12 (corrected with HCl and NaOH) were prepared. 100 μM of DNA stock was prepared in tris-EDTA buffer (pH 8.00) and stored at -20 °C. Working DNA solutions were prepared by diluting the stock solution to the desired concentrations in phosphate buffer solution, stored at 4 °C and not used when older than 4 weeks. Solutions of 6 mM G4 PPI (Molecular mass = 3514 g/mol) and 5 mM HAuCl_4_ were prepared in water.

### 3.3 Equipment and apparatus

A three electrode system was used to perform all electrochemical experiments. A glassy carbon electrode (GCE) with 0.3 cm diameter was used as the working electrode, a platinum wire served as the counter electrode, and Ag/AgCl (3 M Cl^-^) as the reference electrode. All voltammetric experiments were performed on an Epsilon (BASi) electrochemical workstation (LaFayette) with oxidative scan direction except stated otherwise. Square wave voltammetry (SWV) measurements were performed by applying an amplitude of 25 mV and frequency of 15 Hz. Differential pulse voltammetry (DPV) measurements were recorded using pulse amplitude of 50 mV, sample width of 10 msec, pulse period of 200 msec. EIS measurements were recorded with a Zahner IM6ex (Germany), at a perturbation amplitude of 10 mV within the frequency range of 100 kHz to 100 mHz. All solutions for electrochemical measurements were de-aerated by bubbling argon through it for 10 minutes. FE-SEM images were captured using a field emission electron microscope (JEOL- JSM 7500F) fitted with a EDAX CDU Leap Detector.

### 3.4 Electro-preparation of platforms and development of the DNA biosensor

#### 3.4.1 Preparation of GCE/PPI, GCE/AuNP and GCE/PPI-AuNP modified electrodes

For all electro-deposition processes, the GCE was mechanically polished with 0.3 and 0.05 micron alumina powder, rinsed with water and then ultrasonicated in water for 4 minutes. The cleanliness of the surface was verified in PBS with potential range of -100 mV to +650 mV where no peak was expected. GCE/Au-NP was prepared by electrodepositing AuNP on clean GCE surface by cycling the electrode potential from -350 mV to +1000 mV for 10 cycles at 50 mV/s using 2.5 mM aqueous HAuCl_4_ as the electrolyte. GCE/PPI was prepared as described for GCE/AuNP except that 3 mM PPI aqueous solution as electrolyte instead of HAuCl_4_. The preparation of GCE/PPI-AuNP electrode system involved the simultaneous cyclic voltammetric deposition of PPI and AuNP on a clean GCE from an argon-degassed electrolyte consisting of 6 mM PPI and 5 mM HAuCl_4_ in a 1:1 v/v ratio. The GCE/AuNP, GCE/PPI and GCE/PPI-AuNP modified electrodes were rinsed with water and characterized by CV, SWV and EIS; and stored at 4 °C when not in use. However, Screen printed carbon electrode (SPCE) was used as substrate (under the same electro-deposition conditions) for FE-SEM measurements.

#### 3.4.2 Immobilization of probe DNA (GCE/PPI-AuNP/ssDNA) and hybridization with target DNA (GCE/PPI-AuNP/dsDNA)

The GCE/PPI-AuNP/ssDNA nanobiosensor was prepared by dropping a 20 μL solution of 2 μM thiolated single strand probe DNA (or probe ssDNA) on the surface of a previously argon-dried GCE/PPI-AuNP, and leaving it to immobilize for 3 h at 25 °C and then successively rinsing with water and phosphate buffer solution to remove any unbound probe ssDNA. The biosensor was stored at 4 °C when not in use. The biosensor was characterized by voltammetry and EIS in PBS and 5 mM (1:1) ferro/ferricyanide solution Fe(CN)_6_^3-/4-^, respectively.

The bio-recognition experiments, was carried out in PBS (1 mL) containing six different concentrations of complementary ssDNA (target ssDNA) ranging from 0.01 to 5 nM. For each hybridization step (each target DNA concentration), the nanobiosensor (GCE/PPI-AuNP/ssDNA) was immersed in the target ssDNA solution for 45 min at 38 °C. The hybridized biosensor (i.e. GCE/PPI-AuNP/dsDNA) was washed thoroughly with water and phosphate buffer solution to remove unbound target ssDNA before making measurements. The impedimetric responses of the biosensor to the target ssDNA were measured in PBS using Fe(CN)_6_^3-/4-^ as the redox probe. However a single concentration of 0.05 nM complementary DNA was used to investigate the voltammetric response.

## 4. Conclusions

A new method of modifying GCE with poly(propyleneimine) dendrimer and a gold nanoparticle nanocomposite and exploiting their properties for immobilization of ssDNA was developed. The GCE/PPI-AuNP nanocomposite platform exhibited reversible electrochemistry, good conductivity, pH sensitivity and excellent catalytic properties toward Fe(CN)_6_^3-/4-^ redox probe. This DNA biosensor was highly sensitive; to the extent that it was able to amperometrically detect target DNA concentrations as low as 0.05 nM in phosphate buffer solution. Using impedimetric detection techniques, the biosensor had a dynamic linearity of 10^-12^ to 10^-9^ M for target DNA (R^2^ = 0.992), which was an order of magnitude improvement in the limit of detection compared to previous studies that reported a linearity of 10^-11^ to 10^-7^ M [32, 61]. This study also showed that owing to the favourable biomolecular immobilization properties of dendrimers and the possibility of modifying their inner core, the PPI-modified GCE can also be applied in enzyme and antibody biosensors.

## Figures and Tables

**Table 1.** Potential parameters obtained from the response of GCE/PPI to pH in 0.1 M phosphate buffer solution (Figure 4b) using both CV and SWV at 100 mV/s.

**pH**	**Epa (mV)**	**Epc (mV)**	**E°′ (mV)**	**AE (mV)**

	**CV [SWV]**	**CV**	**CV**	**CV**
2.17	388 [340]	336	362	52
4.13	320 [284]	270	295	50
6.17	249 [236]	213	231	36
7.04	223 [216]	201	212	22
8.02	211 [172]	149	180	62
10.16	-[155]	186	-	-
12.00	-	-	-	-

**Table 2.** The EIS parameters obtained from the circuit fitting of plots in Figure 5b.

**Circuit element**	**R_s_(Ω)**	**Ret_ct_(Ω)**	**CPE (nF)**	**Z_W_**
**GCE**	258	1348	463	699
**GCE/PPI-AuNP**	236	251	434	617
**GCE/PPI-AuNP/ssDNA**	212	528	468	604
**Average Error**	6.88	2.97	8.31	2.47

**Table 3.** EIS parameters of GCE/PPI-AuNP/dsDNA obtained from Figure 7a.

**Target DNA conc. (nM)**	**0.01**	**0.05**	**0.1**	**0.5**	**1**	**5**
**log (Target DNA conc. (nM))**	**-11**	**-10.3**	**-10**	**-9.3**	**-9**	**-8.3**
**R_ct_(Ω)**	538.3	651.2	680.5	850.4	908.8	981
**Error (R_ct_)**	0.43	1.24	0.93	0.6	0.36	0.53
**R_s_(Ω)**	127.4	183	182.9	176.4	161.8	167.4
**CPE (nF)**	852	871.3	850.5	802.7	830	812.8
**Z_w_**	301.3	286.2	286.7	288.5	282.9	285.1
